# Comparative Pollen Morphology of Selected Species of *Blumea* DC. and *Cyathocline* Cass. and Its Taxonomic Significance

**DOI:** 10.3390/plants12162909

**Published:** 2023-08-09

**Authors:** Yulan Peng, Xuemei Pu, Qi Yu, Hailing Zhou, Tianfang Huang, Bo Xu, Xinfen Gao

**Affiliations:** Chengdu Institute of Biology, Chinese Academy of Sciences, Chengdu 610041, Chinazhouhl0828@163.com (H.Z.); huangtf@cib.ac.cn (T.H.); xubo@cib.ac.cn (B.X.); xfgao@cib.ac.cn (X.G.)

**Keywords:** *Blumea*, identification, pollen morphology, scanning electron microscopy

## Abstract

The pollen morphology of 20 species from *Blumea* and *Cyathocline* Cass. was investigated using a light microscope (LM) and scanning electron microscopy (SEM) to explore their taxonomic significance. This study showed that pollen grains of these species were usually tricolporate, rarely tetracolporate (*B. sinuata*). Nine pollen types were distinguishable through the exine sculpture characters and the number of apertures. It was easily distinguished *Cyathocline* from species of *Blumea* s. str. by its much smaller size (15.04 μm × 15.07 μm) and sparse and longer spines (24 spines, spine length 4.23 μm) with acute apex, which suggest that *C. purpurea* might not belong to the genus *Blumea* s. str. The palynological characteristics indicated that Section *Macrophllae* and Section *Paniculatae* of *Blumea* were not monophyletic groups. The pollen morphology differentiation of *B. lacera* clade is consistent with the interspecific relationship revealed by the molecular phylogenetic tree. However, the pollen morphology of the *Blumea densiflora* clade is inconsistent with the interspecific relationship based on molecular phylogenetic analysis. This palynology research can only partly support the previously published molecular phylogeny of *Blumea* s. str.

## 1. Introduction

*Blumea* DC. is the largest genus in the tribe Inuleae [1], comprising 50–100 species [2,3,4]. The taxonomy of this genus is notoriously difficult because there is a lack of distinguishing features among species and related genera. Many new species were described and based on limited specimens in the herbaria [4,5,6,7,8,9]. Moreover, a lack of deep morphological study led to errors in taxonomic treatment and great controversy in the classification of *Blumea* [4,5,6,10,11,12]. In recent years, molecular phylogenetic studies supported that *Blumea* was a monophyletic group, excluding *Laggera* Sch. Bip. ex Benth. & Hook. f., *Placus* Lour., *Doellia* Sch.-Bip. and *Blumeopsis* Gagnep. [12,13,14]. Although *Cyathocline* Cass. has long been placed in the tribe Astereae Cass., recent systematic study has found that *Cyathocline purpurea* (Buch.—Ham. ex De Don) O. Kuntze belongs to the genus *Blumea* [15]. The capitula of *C. purpurea* are small, with 2- or 3-seriate phyllaries, pinnatifid leaves and purple florets. The morphological characteristics of *C. purpurea* are very different from other members of the genus *Blumea*. Therefore, the relationship between *Cyathocline* and *Blumea* needs further study.

*Blumea duclouxii* is different from other species of *Blumea* in that they possess the capitula with only one layer of phyllary. Its system position is still uncertain. The genus *Blumea* was established by De Candolle (1833) [16]. The species boundaries are still difficult to delimit. For example, *B. riparia* and *B. megacephala* were treated as two variants under the same species by Randeria [2] and Pornpongrungrueng et al. [4]. While Zhang & Yu [17]. Chen & Anderberg [3] divided them into two different species. De Candolle [5] divided the species of *Blumea* into two series. Randeria [2] interpreted the proposed groups of De Candolle [5] as the sectional rank of *Blumea*. Then sectional classifications were applied and revised by Randeria [2] and Dakshini & Prithipalsingh [18]. However, molecular phyogenetic studies of Pornpongrungrueng et al. [12] were not congruent with those of this genus’s previously published sectional classification. Therefore, more discriminative evidence of these species is needed for the taxonomic revision of the genus *Blumea*.

The mature pollen of plants has unique morphological characteristics, including pollen size, shape, number of apertures, etc., which is of great significance for plant classification and plant origin inference. Pollen morphology sometimes has important taxonomy value in problematic taxa and is widely used in the taxonomic treatment of Asteraceae [19,20,21]. Wortley et al. [20] found that pollen characters may help place rogue genera of Asteraceae. Since Asteraceae pollen was first studied by Fischer [22], several studies of this family were investigated by Wodehouse [23,24,25], Erdtman [26], Stix [27] and Skvarla and Turner [28] by light microscope. Modern research indicated that more subtle morphological differences could be exhibited by electron microscopy [29,30,31,32,33,34]. The study of Reshmi & Rajalakshm showed that palynological characteristics such as aperture type and spine length were significant in the delimitation of taxa in the interspecific levels [29]. The survey of 132 genera and 266 species of the tribe Inuleae by Wittenbach [30] indicated that the tribe Inuleae pollen exhibited a wide diversity. Three pollen types were found in the tribe Inuleae from Egypt [31]. The study of Coutinho & Dinis [32] found that all pollen grains of seven genera and nine species of the subtribe Inulinae had a senecioid pattern of exine, and all quantitative traits had a continuous transition among the different species. It was also found that the exine sculpture characters, especially the spines, were the most useful in defining the genus *Pulicaria* pollen types and distinguishing the species [33].

Until now, only a few species of the genus *Blumea* have been investigated in palynology [30,35,36]. Pollen morphology of two species of *Blumea* s. str. was studied by Wittenbach [30] using the light microscope (LM), including *B. mollis* (D. Don) Merr. (synonym of *B. axillaris* (Lamarck) Candolle) and *B. laciniata* (Roxb.) DC. (synonym of *B. sinuata* (Loureiro) Merrill). LM study of *B. axillaris* found its shape was sub-spheroidal with hetropolar polarity [35]. The pollen morphology of *B. lacera* and *B. obliqua* were similar in both LM and SEM studies [36]. The palynological characteristics of most species in *Blumea* are still unclear.

The systematic development research outlined above indicates that the boundaries of *Blumea* are unclear. Besides the problematic delimitation of the genus itself, the species-level taxonomy of *Blumea* is partly unresolved. This study aims to explore the palynological properties of *Blumea* and the putatively related genus *Cyathocline* and try to estimate their possible taxonomic positions based on pollen morphology.

## 2. Results

### 2.1. Pollen Shape

**Figure 1:**
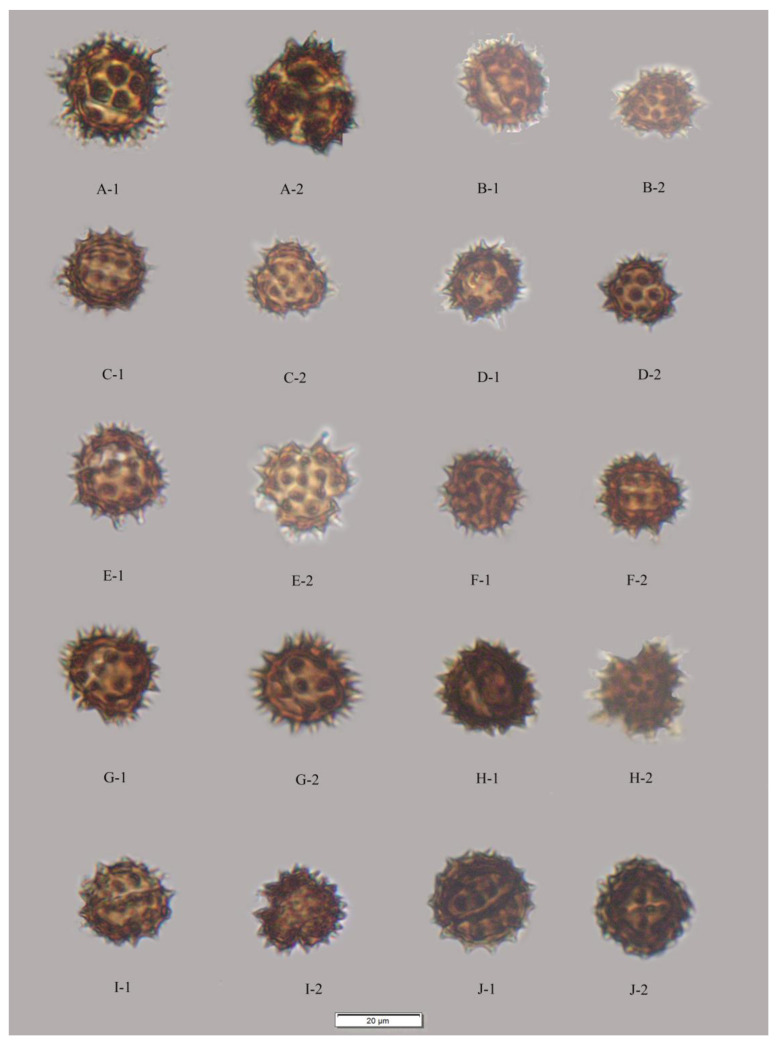
LM micrographs of pollen grains. (**A**) *B. aromatica* (400×), (**A-1**) Polar view, (**A-2**) Equatorial view; (**B**) *B. balsamifera* (400×), (**B-1**) Equatorial view, (**B-2**) Polar view; (**C**) *B. clarkei* (400×), (**C-1**) Equatorial view, (**C-2**) Polar view; (**D**) *Cyathocline purpurea* (400×), (**D-1**) Equatorial view, (**D-2**) Polar view; (**E**) *B. densiflora* (400×), (**E-1**) Equatorial view, (**E-2**) Polar view; (**F**) *B. duclouxii* (400×), (**F-1**) Polar view, (**F-2**) Equatorial view; (**G**) *B. eberhardtii* (400×), (**G-1**) Equatorial view, (**G-2**) Polar view; (**H**) *B. fistulosa* (400×), (**H-1**) Equatorial view, (**H-2**) Polar view; (**I**) *B. hieraciifolia* (400×), (**I-1**) Equatorial view, (**I-2**) Polar view; (**J**) *B. hookeri* (400×), (**J-1**) Equatorial view, (**J-2**) Polar view.

**Figure 2:**
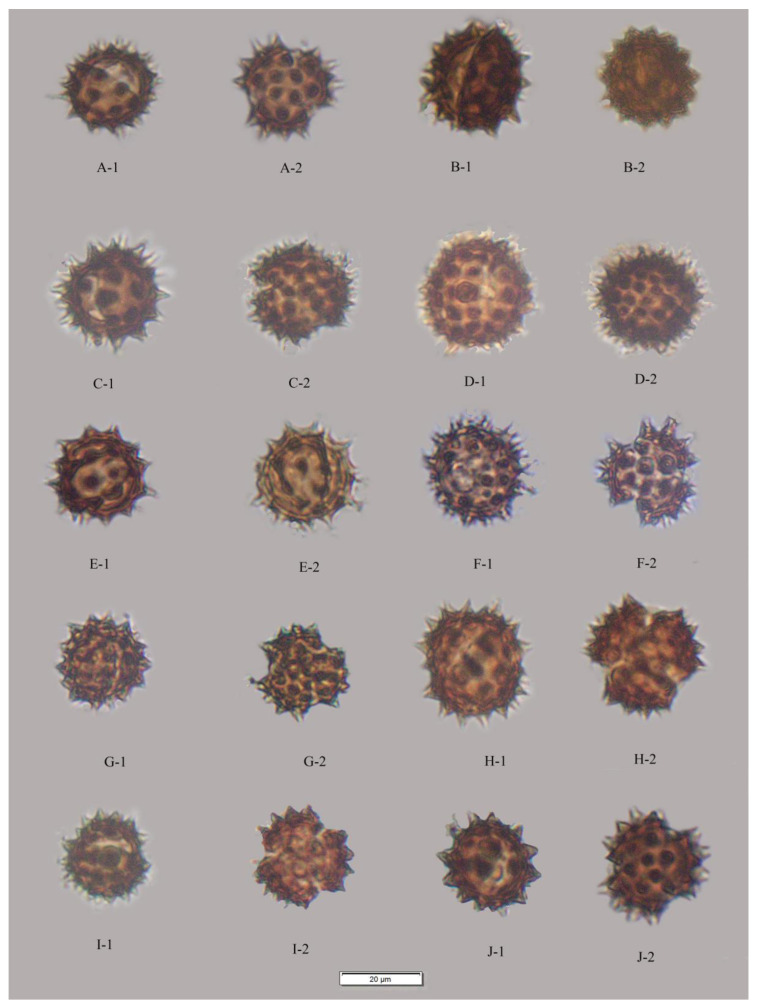
LM micrographs of pollen grains in *Blumea.* (**A**) *B. lacera* (400×), (**A-1**) Equatorial view, (**A-2**) Polar view; (**B**) *B. martiniana* (400×), (**B-1**) Equatorial view, (**B-2**) Polar view; (**C**) *B. megacephala* (400×), (**C-1**) Equatorial view, (**C-2**) Polar view; (**D**) *B. napifolia* (400×), (**D-1**) Equatorial view, (**D-2**) Polar view; (**E**) *B. riparia* (400×), (**E-1**) Equatorial view, (**E-2**) Polar view; (**F**) *B. repanda* (400×), (**F-1**) Equatorial view, (**F-2**) Polar view; (**G**) *B. sessiliflora* (400×), (**G-1**) Equatorial view, (**G-2**) Polar view; (**H**) *B. sinuata* (400×), (**H-1**) Equatorial view, (**H-2**) Polar view; (**I**) *B. paniculata* (400×), (**I-1**) Equatorial view, (**I-2**) Polar view; (**J**) *B. virens* (400×), (**J-1**) Equatorial view, (**J-2**) Polar view.

**Figure 3:**
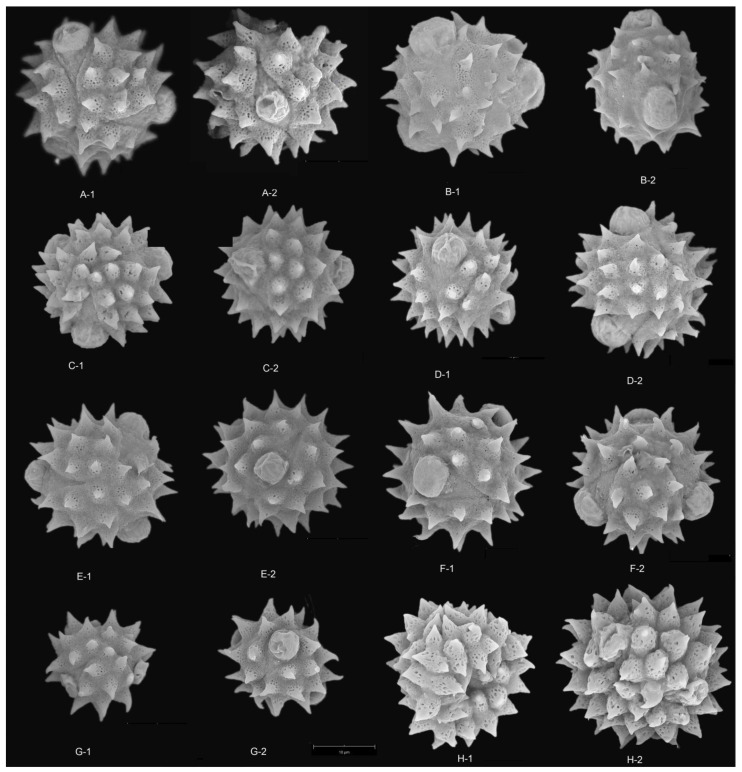
SEM micrographs of pollen grains in *Blumea.* (**A**) *B. aromatica* (8500×), (**A-1**) Polar view, (**A-2**) Equatorial view; (**B**) *B. densiflora* (8500×), (**B-1**) Polar view, (**B-2**) Equatorial view; (**C**) *B. balsamifera* (8400×), (**C-1**) Equatorial view, (**C-2**) Polar view; (**D**) *B. duclouxii* (8500×), (**D-1**) Equatorial view; (**D-2**) Polar view; (**E**) *B. clarkei* (8400×), (**E-1**) Polar view, (**E-2**) Equatorial view; (**F**) *B. eberhardtii* (8400×), (**F-1**) Equatorial view, (**F-2**) Polar view; (**G**) *Cyathocline purpurea* (8400×), (**G-1**) Polar view, (**G-2**) Equatorial view; (**H**) *B. fistulosa* (8500×), (**H-1**) Polar view; (**H-2**) Equatorial view.

**Figure 4:**
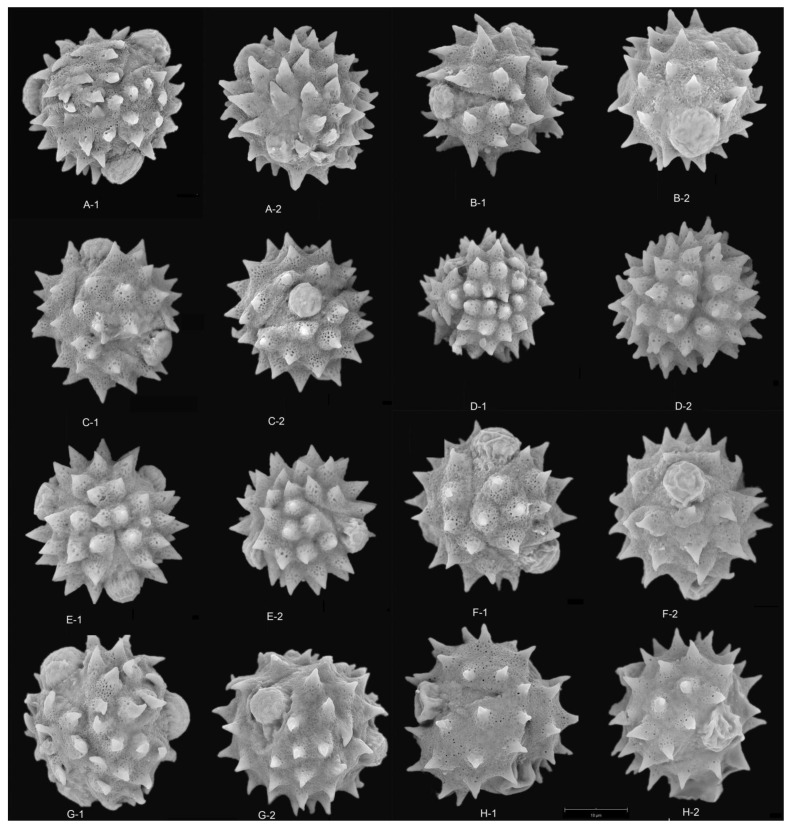
SEM micrographs of pollen grains in *Blumea.* (**A**) *B. hieraciifolia* (8500×), (**A-1**) Polar view, (**A-2**) Equatorial view; (**B**) *B. megacephala* (8500×), (**B-1**) Equatorial view, (**B-2**) Polar view; (**C**) *B. hookeri* (8400×), (**C-1**) Polar view, (**C-2**) Equatorial view; (**D**) *B. napifolia* (8000×), Polar view; (**E**) *B. lacera* (8500×), (**E-1**) Equatorial view, (**E-2**) Polar view; (**F**) *B. riparia* (8500×), (**F-1**) Polar view, (**F-2**) Equatorial view; (**G**) *B. martiniana* (8500×), (**G-1**) Polar view, (**G-2**) Equatorial view; (**H**) *B. repanda* (8400×), (**H-1**) Polar view, (**H-2**) Equatorial view.

**Figure 5:**
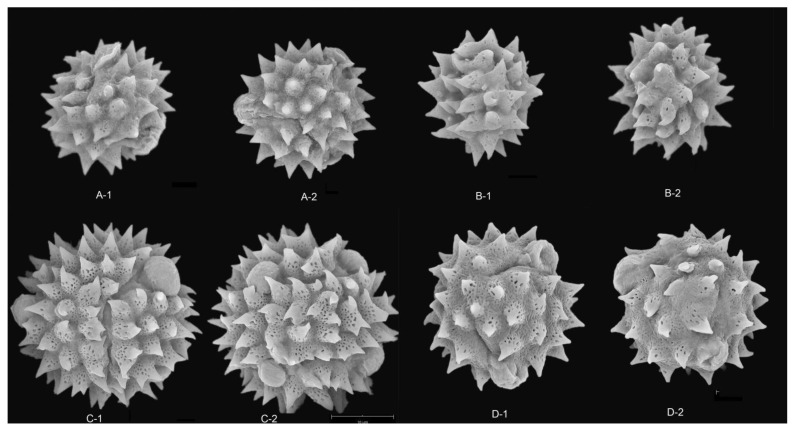
SEM micrographs of pollen grains in *Blumea.* (**A**) *B. sessiliflora* (8400×), (**A-1**) Polar view, (**A-2**) Equatorial view; (**B**) *B. paniculata* (8400×), Polar view; (**C**) *B. sinuata* (8400×), (**C-1**) Polar view, (**C-2**) Equatorial view; (**D**) *B. virens* (8500×), (**D-1**) Equatorial view, (**D-2**) Polar view.

The results showed that the pollen grains of the studied species were radially symmetrical and isopolar, most of these pollen grains were subprolate to spherical and occasionally triangular (Figure 1, Figure 2, Figure 3, Figure 4 and Figure 5), and the ratio of polar axis length to equatorial axis length (P/E) was ranging from 0.97 to 1.11. The measured morphological characteristics of pollen are shown in Table S1. Pollen grains of *Blumea* were 15.07–23.09 μm in equatorial diameter (Table S1). Significant differences existed in the P, E and P/E of pollen between different species (*p* < 0.01).

### 2.2. Apertures

In this study, pollen apertures were tricolporate and tetracolporate [Figure 1, Figure 2 and Figure 5] [Table S2]. The tricolporate was easily observed under both light and scanning electron microscopy. Under SEM, more subtle characters of apertures could be observed. Colpus was usually rather long, not curved, with a narrower end and a wider middle. Excluding pollen grains of *B. sinuata* (Loureiro), Merrill was tetracolporate [Figure 5(C-1,C-2)], and the pollen grains of the other species were tricolporate.

### 2.3. Pollen Exine Ornamentation

All the pollens were echinate. Exines of the pollen grain were microperforate and spinose. Spines were attenuated or contracted in acute or blunt apex. The spines were roughly conical or distinctly broader at their bases and ranged from 2.78–5.23 μm in length (Table S2). There were significant differences in the quantity and length of spines among different species (*p* < 0.01). The exines of pollen grains were microperforate, with round or irregular perforations of varying sizes. Pollens could be roughly divided into two types according to the different densities of interspinular microperforations. The first type was microperforations sparsely distributed in the gap among the base of spines, such as *B. balsamifera* [Figure 3(A-1,A-2)] and *B. repanda* [Figure 4(H-1,H-2)]. The second type was dense microperforations between the spines of all outer walls of pollen, such as *B. martiniana* [Figure 4(G-1,G-2) ] and *B. sinuata* [Figure 5(B-1,B-2)].

### 2.4. Multivariate Analysis

**Figure 6:**
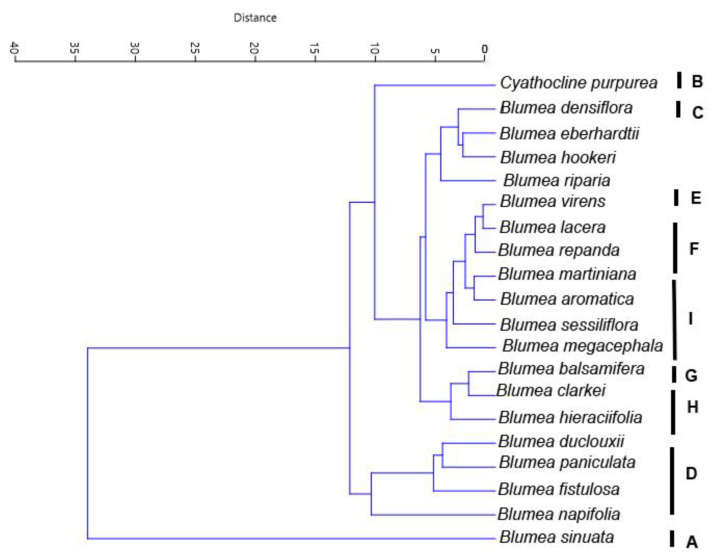
UPGMA Cluster analysis using characteristics of investigated taxa.

For cluster analysis (UPGMA), the pollen characteristics of 20 taxa were analyzed, and their infrageneric and interspecific relationships were observed based on these sampling (Tables S1 and S2, Figure 6). The delimitation of these groups was mainly based on the number of spines, spine length, polar diameter and equator diameter, tip shape of spines and density of interspinular microperforations (*p* < 0.001). In the dendrogram tree (Figure 6), the 20 taxa were divided into four major groups. Except for *Cythocline purpurea* and *Blumea sinuata*, the other groups were divided into four subclusters. *Blumea paniculata* and *B. napifolia* and *B. fistulosa* formed a subcluster (Figure 6).

### 2.5. Pollen Descriptions for the Taxa Studied

#### 2.5.1. *Blumea* Section Macrophllae DC.

The equatorial length of pollen grains ranged from 15.91 µm to 21.41 µm, and the polar axis length ranged from 16.07 µm to 21.37 µm. Pollen grains were spherical or triangular in equatorial view, tricolporate and echinate, with 27–34 spines in the exines. The spines were 2.78 µm to 4.32 µm in length. Exines of the pollen grain were densely microperforate in most species, except sparse inter-spinular perforations in *B. balsamifera*.

Species examined: *Blumea aromatica* [Figure 1(A-1,A-2) and Figure 3(A-1,A-2)], *B. balsamifera* [Figure 1(B-1,B-2,C-1,C-2)], *B. densiflora* (Figure 1(B-1,B-2) and Figure 3(B-1,B-2)], *B. martiniana* [Figure 2(B-1,B-2) and Figure 4(G-1,G-2)], *B. hookeri* [Figure 1(J-1,J-2) and Figure 4(C-1,C-2)].

#### 2.5.2. *Blumea* Section Paniculatae DC.

The equatorial length of pollen grains ranged from 14.47 µm to 23.09 µm, and the polar axis length ranged from 16.03 µm to 23.69 µm. Pollen grains were oblate-spheroidal, spherical in equatorial view, tricolporate or tetetracolporate, and echinate. The polar areas were large, each with 33–79 spines. The spines were roughly conical or distinctly broader at their bases and ranged from 3.37 µm to 5.23 µm in length.

Species examined: *Blumea clarkei* [Figure 1(C-1,C-2) and Figure 3(E-1,E-2)], *B. fistulosa* [Figure 1(H-1,H-2) and Figure 3(H-1,H-2)], *B. hieraciifolia* [Figure 1(I-1,I-2) and Figure 4(A-1,A-2)], *B. lacera* [Figure 2(A-1,A-2) and Figure 4(E-1,E-2)], *B. sinuata* [Figure 2(H-1,H-2) and Figure 5(C1,C-2)], *B.* *duclouxii* [Figure 1F and Figure 3D], *B. napifolia* [Figure 2(D-1) and Figure 4(D-2)], *B. sessiliflora* [Figure 2(G-1,G-2) and Figure 5(A-1,A-2)] and *B. virens* [Figure 1(J-1,J-2) and Figure 5(D-1,D-2)].

#### 2.5.3. *Blumea* Section Semivestitae DC.

The equatorial length of pollen grains ranged from 17.84 µm to 20.77 µm, and the polar axis length ranged from 17.34 µm to 21.71 µm. Pollen grains were spherical, tricolporate and echinate. The spine numbers were 26–33, average 29 (Table S1). The spines were roughly conical or distinctly broader at their bases, ranging from 3.21 µm to 5.75 µm in length, acute or blunt.

Species examined: *B. eberhardtii* [Figure 1(G-1,G-2) and Figure 3(F-1,F-2)], *B. megacephala* [Figure 2(G-1,G-2) and Figure 4(B-1,B-2)], *B. repanda* [Figure 2(F-1,F-2) and Figure 4(H-1,H-2)] and *B. riparia* [Figure 2(E-1,E-2) and Figure 4(F-1,F-2)].

#### 2.5.4. *Cythocline Purpurea*

The equatorial length of pollen grains ranged from 13.15 µm to 16.33 µm, and the polar axis length ranged from 14.14 µm to 17.13 µm. Pollen grains of this species were spherical, tricolporate and echinate. The polar areas were large, each with 21–28 spines, an average of 24. Spines are conical, acute, 3.46–4.56 μm in length, average 4.23 μm. The tips of the spines were sharp.

Specimen examined: SE01957, SE02153 [Table 1, Figure 1(D-1,D-2) and Figure 3(G-1,G-2)].

### 2.6. Pollen Types Description

According to the comparative study of pollen morphology, the pollen of all these species could be divided into nine categories, i.e., *Blumea sinuata* pollen type, *Cythocline purpurea* pollen type, *B. densiflora* pollen type, *B. napifolia* pollen type, *B. aromatica* pollen type, *B. virens* pollen type, *B. repanda* pollen type, *B. balsamifera* pollen type and *B. clarkei* pollen type. A brief description of each pollen type was as follows:

Type A *Blumea sinuata* pollen type

The typical characteristics of this kind of pollen were tetracolporate, spherical, with denser and longer spines. The pollen grain size was large, 19.52–23.90 μm in equatorial diameter, and the polar axis was 19.72–24.30 μm. This pollen was only found in *B. sinuata* [Figure 5(C-1,C-2)].

Type B *Cythocline purpurea* pollen type

Pollen size of this type was much smaller (13.15–16.33 µm × 14.14–17.13 µm), tricolporate, spherical, with sparse and longer spines (number of spines < 30, the average length of spine > 4 µm), dense interspinular perforations. The tips of the spines were sharp. This type of pollen was only found in *C. purpurea* [Figure 3(G-1,G-2)].

Type C *Blumea densiflora* pollen type

This type of pollen was triangular or spherical, with sparse (the number of spines < 33) and short spines (the length of spines < 3 µm). This type of pollen was only found in *B. densiflora* (Figure 4A).

Type D *Blumea napifolia* pollen type

The typical characteristics of this kind of pollen were tricolporate, spherical, with denser and longer spines. This type was similar to *B. sinuata* type, except it was tricolporate. This kind of pollen was found in *B. napifolia* [Figure 4(D-1,D-2)], *B. fistulosa* [Figure 3(H-1,H-2)] and *B. paniculata* [Figure 5(B-1,B-2)]. The pollen grain size of *B. paniculata* was smaller than that of *B. napifolia* and *B. fistulosa*.

Type E *Blumea virens* type

This type was similar to *B. aromatica* type, but the grooves of apertures were deeper than those of *B. aromatica* type. This pollen was only found in *B. virens* [Figure 5(D-1,D-2)].

Type F *Blumea repanda* pollen type

The typical characteristics of this kind of pollen were tricolporate and spherical, with longer and sharp spines and relatively dense inter-spinular perforations. This type of pollen was found in *B. repanda* [Figure 4(H-1,H-2)] and *B. lacera* [Figure 4(E-1,E-2)].

Type G *Blumea balsamifera* pollen type

This type of pollen was spherical, tricolporate, with a medium number of spines. Pollen grains were trifid in polar view. Spines were short, with a blunt apex. Exines were relatively sparse microperforate.

Type H *Blumea clarkei* pollen type

This type of pollen was spherical, tricolporate, with a medium number of spines. Pollen grains were trifid in polar view. Spines were long, with acute apex. Exines were relatively sparse microperforate. This type of pollen was found in *B. hieraciifolia* [Figure 4(A-1,A-2)] and B. *clarkei* [Figure 4(E-1,E-2)].

Type I *Blumea aromatica* pollen type

This type of pollen was spherical, tricolporate, with a medium number of spines. Pollen grains were trifid in polar view. Exines were relatively dense microperforate, with sparse spines. Spines were short and blunt. Most species had this pollen type. We found this type of pollen in *B. hookeri* [Figure 4(B-1,B-2)], *B. martiniana* [Figure 3(D-1,D-2)], *B. riparia* [Figure 4(F-1,F-2)] and *B. megacephala* [Figure 4(B-1,B-2)].

See the following key for a comparison of different pollen types.

## 3. Discussion

All the studied pollen grains had common characteristics such as spines and inter-spinular perforations on their exines, which were nearly spherical and were typical types of pollen grains of the tribe Inuleae, some similar to that of the other entomophilous Inuleae species such as *Pulicaria* and *Inula* [31,32,33], indicating a close genetic relationship between these species. The pollen morphology of *Blumea* was similar to those of *Blumea* species of Wittenbach [30]. In this study, pollen showed significant interspecific variation in size and exine spines.

### 3.1. Size Range

Among all the studied species, the pollen grains were highly variable in size. Although pollen size of Inuleae species had not been used as a primary factor for delimitation of pollen types alone, because of pollen grain size possibly related to polyploidy within a species of Asteraceae [37,38,39,40]. The environmental and nutritional conditions, as well as the processing methods of pollen, also affected the size of the pollen grain [41]. The *Blumea sinuata* type pollen was quite different from the other species. This species was also significantly different from the allied species in that the stem of *B. sinuata* was noticeably stouter. It was possibly related to the polyploidy of this species, according to the chromosome number counts. But under similar environmental conditions and the same treatment method for pollen grains, there were still significant differences in pollen size among some species. Of all the species studied, *Cythocline purpurea* has the smallest pollen grains.

### 3.2. Apertures

The aperture structure also contributed to differentiating *Blumea sinuata* from the other investigated taxa, which was characterized by both 3-zonocolporate and 4-zonocolporate pollen grains, while the other taxa exhibited only 3-zonocolporate pollen grains. The tetracolporate form pollen was unusual in the Asteraceae, although this type had been reported by Wodehouse [25], Wittenbach [30], Osman [31], and Wortley [20]. Wodehouse [25] believed that tetracolporate aperture, perhaps, was related to the irregular distribution of chromosomes or as the result of hybridity. Among all studied species, only *B. sinuata* was tetracolporate. This was also possibly related to its polyploidy.

### 3.3. Exine Sculpturing

The pollen sculpture was somewhat uniform in most investigated species. The pollen grains were spiny with perforate sculpture. In this study, the pollens examined were highly variable regarding the number and shape of exine spines. Although the tendency toward spine reduction of Inuleae was considered an advancement character in the Asteraceae family [23], we believed that the high number of spines in *Blumea sinuata* pollen was possibly related to its polyploidy. Spine length and density were stable morphological characters for generic differentiation [23,25]. Among all investigated species, spine length, density and shape could also be used as species delimitation. The inter-spinular perforation density varied among different species (Table S2). Compared to other species, the pollen of *Cythocline purpurea* had the least number of spines and longer spines.

### 3.4. Taxonomic Significance of Pollen Features

Three pollen types could be found in *Blumea* Section *Macrophllae* DC., i.e. *B. densiflora* type, *B. balsamifera* type and *B. aromatica* type. The pollen grains of *B. densiflora* were smaller, triangular or spherical, with the shortest and least spines, and were quite easily distinguished from the other species in this section. The pollen grains of most species in this section were spherical, occasionally, with few triangular pollen in *B. martiniana*. The palynological characteristics of other species in the Section *Macrophllae* had little variation, except in pollen grain size and the number and length of spines and microperforations of exines. The pollen grains of *B. balsamifera* were the smallest, with the most exine spines in this section and sparsely inter-spinular microperforations. The pollen grain of *B. hookeri* had the longest spines in this section, followed by *B. aromatica.* Although *B. hookeri* and *B. densiflora* were treated as one species by some botanist, the pollen morphological characteristics were significantly different.

The palynological characteristics of species in Section *Paniculatae* DC. varied greatly. The pollen grains were tricolporate or tetracolporate. The pollen of *Blumea sinuata* was different from that of the other species, with four germination holes, the greatest number of spines and the longest spines. The pollen grains of *B. napifolia* and *B. fistulosa* were larger, with more and longer spines. The pollen morphological characters of *B. paniculata* were similar to those of *B. napifolia* in that polar region fissures were not obvious, and spines were denser. But the pollen grain size was smaller than that of *B. napifolia. Blumea duclouxii* had the smallest pollen grains in this section, followed by *B. lacera*. The palynological characteristics of *B. duclouxii* were very similar to those of *B. lacera*. It was difficult to decide its taxonomic position only by pollen morphology.

The morphological characteristics of all the species studied in *Blumea* Section *Semivestitae* were relatively consistent, except for slightly larger pollen grains and the relatively sparse microperforate exines with longer acute spines in *B. repanda*. Pollen morphological characteristics of *B. riparia* and *B. megacephala* in Section *Semivestitae* were similar to that of *B. aromatica*, *B. hookeri*, *B. martiniana* in Section *Macrophllae*.

From the pollen characteristics of all studied species, we can find that the palynological characteristics of species do not support the taxonomic classification of the sections of *Blumea* De Candole [5]. The pollen morphology of different sections may be consistent. On the other side, the division of the sections of *Blumea* was artificial by various authors [2,5,18]. *Blumea* Section *Macrophllae* and Section *Paniculatae* were probably not monophyletic groups, with different evolutionary directions and variable pollen morphology. However, pollen characteristics have a certain significance in the species identification of *Blumea*. For example, pollen grains of *B. sinuata* were tetracolporate, while pollen grains of *B. densiflora* pollen were triangular, sparsely microperforate. However, species with similar pollen types were difficult to segregate.

*Cythocline purpurea* had the smallest pollen grains, with the least spines, of all the species studied. Spines were long, with acute apices in *C. purpurea.* This combination of characters was quite different from the other studied species. The pollen morphological characters of *C. purpurea* were easily distinguished from other species of *Blumea* s. str., which suggested that *C. purpurea* might not belong to the genus *Blumea s*. str.

Two major well-supported clades of *Blumea s. str*. were recognized based on a molecular phylogenic tree by Pornpongrungrueng et al. [12], including *Blumea densiflora* clade and *B. lacera* clade. Our study showed polymorphism in the pollen morphology of the *B. densiflora* clade and the *B. lacera* clade. The pollen morphology differentiation of the *B. lacera* clade was consistent with the interspecific relationship revealed by the molecular phylogenetic tree. For example, *B. napifolia* and *B. paniculata* were closely related in the molecular tree and showed similar pollen characteristics. But pollen morphology of *B. densiflora* clade, including *B. densiflora*, *B. aromatica*, *B. balsamifera* and *B. martiniana* displayed significant variation, which is inconsistent with the interspecific relationship suggested by the molecular morphology tree [12]. Therefore, our palynology research can only partly support the previously published molecular phylogeny of *Blumea* s. str.

## 4. Methods and Materials

### 4.1. Plant Materials

Mature pollen samples were obtained by removing one or two florets from dried specimens. Voucher specimens were deposited in the Herbarium of Chengdu Institute of Biology (CDBI). The herbarium voucher details were included in the specimens investigated list (Table 2).

### 4.2. Micromorphological Examination

Pollen samples were prepared by acetolysis as described by Erdtman [42] and viewed with a light microscope (LM). Pollen mounted in neutral gum was examined under transmission light using Olympus microscopes BX43. Pollen samples were prepared for the scanning electron microscope (SEM). Pollen grains were dried in the air, then directly mounted on stubs and sputter-coated with gold-palladium for five minutes. SEM examination was carried out by Phenom Pro microscope, operating at 10 kV. Images were digitally processed, and the final plates were prepared using Adobe PhotoShop 7.0. Pollen characteristics, including polar length of pollen grains (P), and equatorial length (E), were measured using digital light microscopy images based on 20 pollen grains from every investigated species by software Image J [43]. Due to the small size of the pollen in the genus *Blumea*, the length and quantity of spines were observed and measured more accurately under scanning electron microscopy. The ratio of polar length to equatorial length (P/E) of pollen was calculated. Pollen morphology was described according to the standards of Erdtman [44] and Wang [45]. If the pollen shape index (P/E) > 2, the pollen was considered perprolate. If 1.32 < P/E ≤ 2, the pollen was considered prolate. If 1.14 < P/E ≤ 1.32, the pollen was considered as subprolate. If 0.88 < P/E ≤ 1.14, the pollen was considered spherical. Descriptive terminology follows Punt et al. [46].

### 4.3. Statistical Analysis

Statistical analysis was performed using PAST version 4.04 for Windows software [47]. For each pollen morphological character (7 quantitative values), a one-way ANOVA technique was used to identify the statistical significance of differences in mean values among the taxa studied. A cluster analysis method-based UPGMA tree was constructed to examine the relationship among the taxa based on pollen characters of 20 species. After assigning quality traits of all pollen, they were used for clustering analysis along with quantitative traits.

## 5. Conclusions

In conclusion, nine pollen types were found in all studied species. The pollen morphology did not support an assumption that *Cyathocline* Cass. was a member of *Blumea*. The palynological characteristics suggested that Section *Macrophllae* and *Paniculatae* of *Blumea* were not monophyletic groups. The pollen morphology of different sections could be consistent but was variable in the same section. Pollen characteristics have a certain significance for the species identification of *Blumea*. Our palynology research can only partly support the previously published molecular phylogeny of *Blumea* s. str.. In the future, it is necessary to conduct palynological studies on more species of this genus and allied genera and other morphological studies, such as the structural characteristics of leaf epidermal cells, which may help to understand the systematic classification of *Blumea.*

## Figures and Tables

**Table 1:** Key to the pollen types delimited in this study.

1. Pollen tetracolporate	*Blumea sinuata* pollen type
–Pollen tricolporate	2
2. Pollen size small (10.51–14.86 µm × 12.01–16.37 µm), spine’s length 3.46 µm to 4.56 µm	
spines number 21–28	*Cythocline purpurea* pollen type
–Pollen size larger (13.71–23.90 µm × 12.53–26.67 µm), spine’s length 2.31 µm to 5.75 µm, spines number 27–58	3
3. Pollen grain triangular, spines short and sparse, spine’s length 2.29–3.43 µm, spines number 20–31	*Blumea densiflora* pollen type
–Pollen is nearly spherical, occasionally triangular, spine length 3.0–5.2 µm	4
4. Spines number 41–49	*Blumea napifolia* pollen type
Spines number 23–37	5
5. Grooves of apertures are deep	*Blumea virens* pollen type
–Grooves of apertures are shallow	6
6. Exines with sparse interspinular microperforations	7
–Exines with dense interspinular microperforations	9
7. Spines long with an acute apex	*Blumea repanda* pollen type
–Spines short with a blunt apex	8. *Blumea balsamifera* pollen type
9. Spines long with an acute apex	*Blumea clarkei* pollen type
–Spines short with a blunt apex	10. *Blumea aromatica* pollen type

**Table 2:** Specimens investigated.

Species	Collection Number	The Individual Number	Collection Site	Collection Date
*Blumea aromatica* DC.	SE02118	3	Yunnan, China	11 January 2019
*Blumea aromatica*	SE02485	3	Yunnan, China	21 January 2019
*Blumea balsamifera* (L.) DC.	SE001856	3	Lam Dong, Vietnam	2 April 2018
*Blumea balsamifera*	SE01483	3	Kontum, Vietnam	26 March 2018
*Blumea clarkei* Hook. f.	SE01183	3	Ninh Binh, Vietnam	21 March 2018
*Blumea densiflora* DC.	SE00759	3	Cao Bang, Vietnam	10 March 2018
*Blumea densiflora*	SE01644	3	Kontum, Vietnam	31 March 2018
*Blumea duclouxii* Vaniot	SE01407	3	Ninh Binh, Vietnam	25 March 2018
*Blumea duclouxii*	SE01426	3	Quang Nam, Vietnam	26 March 2018
*Blumea eberhardtii* Gagnep.	SE02259	3	Yunnan, China	14 January 2019
*Blumea eberhardtii*	SE02448	3	Yunnan, China	20 January 2019
*Blumea fistulosa* (Roxb.) Kurz	SE01055	3	Ha Giang, Vietnam	16 March 2018
*Blumea fistulosa*	SE01975	3	Yunnan, China	6 January 2019
*Blumea hieraciifolia* (Sprengel) Candolle	SE02423	3	Yunnan, China	17 January 2019
*Blumea hookeri* C. B. Clarke ex Hook. f.	SE00934	3	Ha Giang, Vietnam	14 March 2018
*Blumea hookeri*	SE02414	3	Yunnan, China	17 January 2019
*Blumea lacera* (Burm. F.) DC.	SE001023	3	Ha Giang, Vietnam	15 March 2018
*Blumea lacera*	SE01022	3	Ha Giang, Vietnam	15 March 2018
*Blumea martiniana* Vaniot.	SE02327	3	Yunnan, China	16 January 2019
*Blumea martiniana*	THP-KD-2601	3	Yunnan, China	7 January 2019
*Blumea megacephala* (Randeria) Chang et Tseng	SE002279	4	Yunnan, China	16 January 2019
*Blumea megacephala*	SE01151	3	Ninh Binh, Vietnam	19 March 2018
*Blumea napifolia* DC.	SE00117	4	Bolikhamxai, Laos	20 January 2018
*Blumea napifolia*	SE01269	3	Ninh Binh, Vietnam	22 March 2018
*Blumea paniculata* (Willd.) M. R. Almeida	SE02007	4	Yunnan, China	7 March 2019
*Blumea paniculata*	SE01962	3	Yunnan, China	6 March 2019
*Blumea repanda* (Roxb.) Hand.-Mazz.	SE02245	3	Yunnan, China	14 January 2019
*Blumea repanda*	SE02278	3	Yunnan, China	16 January 2019
*Blumea riparia* (Bl.) DC.	SE00875	3	GiangYen Minh, Vietnam	13 March 2018
*Blumea riparia*	SE001243	3	Ninh Binh Nho Quan, Vietnam	21 January 2018
*Blumea sessiliflora* Decne.	SE00547	3	Yunnan, China	17 January 2019
*Blumea sessiliflora*	SE01956		Yunnan, China	6 January 2019
*Blumea sinuata* (Loureiro) Merrill	SE01092	3	Laocai, Vietnam	16 March 2018
*Blumea sinuata*	SE02272	3	Yunnan, China	15 January 2019
*Blumea virens* DC.	SE0154	3	Bolikhamxai, Laos	20 January 2018
*Blumea virens*	SE0148	3	Bolikhamxai, Laos	20 January 2018
*Cyathocline purpurea* (Buch.-Ham. ex De Don) O. Kuntze.	SE01957	3	Yunnan, China	6 January 2019
*Cyathocline purpurea*	SE02153	3	Yunnan, China	6 January 2019

## Data Availability

The datasets used and analyzed during the current study are available from the corresponding author upon reasonable request.

## Supplementary Materials

The following supporting information can be downloaded at: https://www.mdpi.com/article/10.3390/plants12162909/s1, Table S1: Summary showing the pollen grains dimensions (LM) in *Blumea* and *Cyathocline*; Table S2: Pollen characters for the species examined in this study by SEM (only great variable characters are shown).
