# Supplementary material for: Comparative Pollen Morphology of Selected Species of Blumea DC. and Cyathocline Cass. and Its Taxonomic Significance
Source: Plants (Basel). 2023 Aug 9;12(16):2909. doi: 10.3390/plants12162909 (PMC10459363; doi:10.3390/plants12162909)
Supplement: Supplementary file 1 [file plants-12-02909-s001.zip › plants-2332344-supplementary.pdf]

Table S1. Summary showing the pollen grains dimensions (LM) in *Blumea* and *Cyathocline*.

Species	P (μm) (Meant±SD, range)	E (μm) (Meant ±SD, range)	P/E (Meant±SD, range)	P*E (Meant±SD, range)	Polar grain shape
Blumea aromatica	20.81±1.44 (18.59–23.66)	21.41±1.46 (18.59–23.31)	0.97±0.05 (0.91–1.09)	329.55±56.83 (356.17–545.91)	Spherical
Blumea balsamifera	16.70±0.91 (14.65–18.59)	15.91±1.19 (14.09–19.16)	1.05±0.06 (0.90–1.12)	231.50±32.28 (222.22–356.13)	Spherical, few triangular
Blumea clarkei	17.43±0.89 (14.65–18.59)	17.15±1.24 (13.75–19.12)	1.0±0.06 (0.89–1.20)	299.60±33.08 (227.26–356.19)	Spherical
Blumea densiflora	18.24±1.12 (16.34–20.2)	18.09±1.19 (16.33–20.00)	1.01±0.05 (0.93–1.08)	300.83±39.13 (266.85–405.64)	Spherical, triangular
Blumea duclouxii	16.03±1.87 (12.53–19.43)	14.47±1.85 (11.50–18.29)	1.11±0.15 (0.89–1.39)	232.00±46.6 (180.37–323.69)	Spherical, few triangular
Blumea eberhardtii	17.67±1.08 (16.06–19.12)	17.53±1.05 (15.94–19.44)	1.01±0.05 (0.87–1.11)	310.37±33.33 (260.27–366.83)	Spherical
Blumea fistulosa	17.09±0.94 (15.21–18.87)	15.94±1.24 (14.65–18.59)	1.08±0.07 (0.95–1.17)	237.33±32.12 (222.81–350.89)	Spherical
Blumea hieraciifolia	18.38±1.10 (17.18–20.85)	17.28±1.54 (14.65–20.85)	1.07±-0.06 (0.98–1.21)	289.90±46.79 (260.00–434.51)	Spherical
Blumea hookeri	19.87±1.41 (16.73–22.82)	18.74±1.21 (16.74–21.11)	1.06±0.04 (1.00–1.16)	373.64±48.25 (280.03–466.83)	Spherical
Blumea lacera	19.72±1.13 (17.53–21.13)	19.03±1.02 (16.90 –21.1)	1.04±0.05 (0.96–1.16)	326.97±37.19 (290.41–440.39)	Spherical
Blumea martiniana	21.37±0.94 (19.72–22.82)	20.13±1.05 (18.27–22.31)	1.06±0.06 (0.95–1.13)	430.33±32.00 (366.60 –488.79)	Spherical
Blumea megacephala	21.71±1.10 (19.16–23.66)	20.77±1.22 (18.72–22.25)	1.05±0.04 (0.99–1.17)	451.79±45.63 (361.51–514.18)	Spherical
Blumea napifolia	23.69±1.38	23.09±1.30	1.03±0.03	476.85±60.57	Spherical

	(21.13–25.92)	(21.11–25.07)	(0.99 –1.08)	(441.60–664.31)	
Blumea riparia	18.42±0.83<br>(16.53-19.62)	17.84±1.43<br>(15.74-20.56)	1.04 -0.08<br>(0.86–1.14)	328.97±33.93<br>(276.65-393.88)	Spherical
Blumea repanda	20.00±1.40<br>(16.93–22.25)	20.06±1.25<br>(17.47–21.69)	1.00±0.05<br>(0.89–1.07)	350.00±48.84<br>(295.23–482.81)	Spherical
Blumea sessiliflora	17.63±1.05<br>(15.49–19.44)	16.75±1.28<br>(13.15–18.31)	1.06±0.05<br>(0.98–1.18)	257.71±37.43<br>(204.24–330.09)	Spherical
Blumea sinuata	22.27±1.30<br>(19.72 –24.30)	21.91±1.58<br>(19.52–23.90)	1.02±0.05<br>(0.93–1.10)	425.75±60.67<br>(408.50–609.88)	Spherical
Blumea paniculata	18.32±1.10<br>(16.90–21.13)	17.84±0.97<br>(16.73–20.56)	1.03±0.04<br>(0.94–1.14)	297.86±36.30<br>(283.31–434.43)	Spherical, few triangular
Blumea virens	20.43±1.41<br>(18.03–22.82)	19.66±1.40<br>(17.18–21.41)	1.04±0.06<br>(0.96–1.17)	403.15±52.43<br>(350.41–476.57)	Spherical
Cyathocline purpurea	15.64±0.80<br>(14.14 - 17.13)	15.07±0.97<br>(13.15 - 16.33)	1.04±0.04<br>(0.96 - 1.11)	236.24±25.72<br>(185.94 - 279.78)	Spherical

Note: All the data are measured in LM (400×), P=Polar axis, E=Equatorial diameter.

Table S2. Pollen characters for the species examined in this study by SEM (only great variable characters are shown).

Species	Number of aperture	Spine's length (μm) (Mean, range)	Spines number (Mean, range)	The apices of spines	Interspinular microperforations	Pollen grain shape
Blumea aromatica	3	4.15 (3.61–4.86)	31 (29–32)	blunt	dense	spherical
Blumea balsamifera	3	3.29 (2.71–4.18)	34 (31–37)	blunt	sparse	spherical
Blumea clarkei	3	4.25 (3.83–4.70)	34 (30–37)	acute	dense	spherical
Blumea densiflora	3	2.78 (2.09–3.43)	27 (20–32)	acute	dense	triangular，spherical
Blumea duclouxii	3	3.37 (3.0–3.4)	39 (33–43)	acute	dense	spherical, few triangular
Blumea eberhardtii	3	4.23 (4.05–4.57)	29 (28–30)	acute	sparse	spherical
Blumea fistulosa	3	4.85 (4.22–5.39)	44 (41–49)	blunt	dense	spherical
Blumea hieraciifolia	3	3.79 (3.39–4.19)	34 (25–42)	blunt	dense	spherical
Blumea hookeri	3	4.38 (4.20–4.61)	29 (26–30)	blunt	dense	spherical
Blumea lacera	3	4.46 (3.47–5.5)	33 (29–37)	acute	sparse	spherical
Blumea martiniana	3	3.75 (3.22–4.46)	32 (28–40)	blunt	dense	spherical
Blumea megacephala	3	4.25 (3.68–5.22)	29 (26–30)	blunt	dense	spherical
Blumea napifolia	3	4.72 (3.57–5.30)	44 (39–58)	blunt	sparse	spherical
Blumea riparia	3	3.93 (3.21–4.35)	26 (23–35)	blunt	dense	spherical
Blumea repanda	3	4.52 (4.03–5.75)	33 (31–36)	acute	sparse	spherical
Blumea sessiliflora	3	3.20 (2.51-3.48)	34 (28–37)	blunt	sparse	spherical
Blumea sinuata	3	5.23 (4.47–6.13)	67 (62–79)	acute	dense	spherical
Blumea paniculata	3	3.91 (3.33-4.41)	40 (35–44)	blunt	dense	spherical
Blumea virens	3	4.07 (3.60–4.64)	33 (30–36)	blunt	dense	spherical
Cyathocline purpurea	3	4.23 (3.46–4.56)	24 (21–28)	acute	dense	spherical

Note: All the values calculated based on the measured mean value magnified by 8400 times.
